# Letter from Chicago

**Published:** 1887-09

**Authors:** Harold N. Moyer

**Affiliations:** 434 W. Adams Street, Chicago


### LETTER FROM CHICAGO.

THE EXCITEMENT ATTENDING THE BOODLE TRIALS—SOME PROMINENT MEMBERS OF THE MEDICAL PROFESSION CONNECTED WITH THE TRIALS—THE NEW MEDICAL PRACTICE ACT—BENEFITS ARISING PROM IT, ETC.

*Editors Atlanta Medical and Surgical Journal:*

The dullness in medical circles during the present heated term has been only partially relieved by the general excitement attending the famous "boodle" trials and the *quasi* connection of some more or less prominent members of the medical profession therewith. The recent arrest of a prominent physician, for direct complicity in the escape of one of the chief offenders, only adds emphasis to the advice that the average physician should avoid the "combines," "deals," "rings" and other inventions of the practical politician. As a matter of fact, the administration of county affairs is so intimately concerned with the expenditure of money for the care of the sick, poor and defective classes that a physician to the "ring" or "combine" has come to be quite an essential feature. They are not usually appointed for their medical knowledge or political acumen, but solely on the degree of faithfulness shown their political employers. Their rewards are at the most a few hundred dollars or some paltry hospital appointment. When one of these "deals" is investigated, the physician, as in the present case, usually comes out at the attenuated end of the horn.

At the last moment, and from the very jaws of defeat, Dr. Rauch, Secretary of the State Board of Health, snatched a substantial victory in the passage of the new medical practice act. The old law has worked well under the energetic administration of the State Board. Under it the percentage of non-graduates has been reduced from 51.4 in 1877 to 7.7 in 1887. All the non-graduates have now been in practice twenty years or more. It will be seen how soon the ten-year clause of the old bill, and which was thought at the time to almost nullify its most essential provision, has become imperative. In addition the board have driven hordes of itinerants and quacks beyond the State, and the whole number of practitioners has been reduced from 7,400 to 6,180 during the past ten years. It may be safely stated that the board cut off at least 2,000 pretentious charlatans that would now disgrace the profession but for the old law and its energetic enforcement.

The present law contains many of the provisions of the old; the board are authorized to grant certificates (without which it is unlawful to practice) to those having diplomas from legally chartered medical institutions in *good standing*. This somewhat vague phrasing is supplemented in the next section (§2) by the following: "It shall issue certificates to all who furnish satisfactory proof of having received diplomas or licenses from legally chartered medical institutions *in good standing as may be determined by the board.*"

It is manifest that under this section very wide discretionary power is vested in the board. It would even seem to be possible to require every one, whether in possession of a diploma or not, to submit to an examination by the board. It is to be regretted that the law did not at once and for all time do away with the "diploma fetich," and substitute a vigorous examination as the sole condition of obtaining a license. It is sufficient to say now, but it is more than probable that this discretionary power will be sustained by the Supreme Court.

Section 10 provides that persons shall be regarded as practicing medicine, within the meaning of the Act, "who shall treat, operate on, or prescribe for any physical ailment of another afflicted." It will be seen that this broad provision is intended to reach the minor forms of quackery, "faith-healing," "metaphysics," "magnetism," etc. Incidentally the physician now has it in his power to cut off that little perquisite of the druggist, "counter prescribing."

These are the essential features of the new law, though some additional features have been added regarding dues, fines and their collection, etc. Itinerants are required to pay a license of one hundred dollars per month. At the last meeting of the board eight applications for this license were refused, and this will probably be the policy of the board in the future.

At the last regular meeting the board decided that all medical colleges, to be considered "in good standing," shall require three full years of study and three courses of medical lectures. The policy of making haste slowly will, we think, result in the best speed in this case as in many others.

The special societies have all adjourned until October. The Chicago Medical manages to hold its two weekly (excuse the pun) meetings, but with numbers reduced to a baker's dozen, and interest in precise proportion to the temperature.

Very truly yours, HAROLD N. MOYER.

*434 W. Adams Street, Chicago, August 2, 1887.*

